# Case report: Severe rhabdomyolysis and acute liver injury in a high-altitude mountain climber

**DOI:** 10.3389/fmed.2022.917355

**Published:** 2022-08-08

**Authors:** Yun-Chih Yeh, Chien-Chou Chen, Shih-Hua Lin

**Affiliations:** ^1^Department of General Medicine, Tri-Service General Hospital, National Defense Medical Center, Taipei, Taiwan; ^2^Department of Internal Medicine, Tri-Service General Hospital Songshan Branch, National Defense Medical Center, Taipei, Taiwan; ^3^Division of Nephrology, Department of Medicine, Tri-Service General Hospital, National Defense Medical Center, Taipei, Taiwan

**Keywords:** acute high-altitude illness, hypobaric hypoxia, rhabdomyolysis, acute kidney injury, abnormal liver function

## Abstract

Concurrent severe rhabdomyolysis and acute liver damage are rarely reported in the setting of acute high-altitude illness (AHAI). We described a 53-year-old healthy mountain climber who experienced headache and dyspnea at the summit of Snow Mountain (Xueshan; 3,886 m above sea level) and presented to the emergency room with generalized malaise, diffuse muscle pain, and tea-colored urine. His consciousness was alert, and he had a blood pressure of 114/74 mmHg, heart rate of 66/min, and body temperature of 36.8°C. Myalgia of the bilateral lower limbs, diminished skin turgor, dry oral mucosa, and tea-colored urine were notable. Urinalysis showed positive occult blood without red blood cells. The most striking blood laboratory data included creatine kinase (CK) 33,765 IU/L, inappropriately high aspartate aminotransferase (AST) 2,882 IU/L and alanine aminotransferase (ALT) 2,259 IU/L (CK/AST ratio 11.7, CK/ALT ratio 14.9), creatinine 1.5 mg/dl, serum urea nitrogen (BUN) 26 mg/dl, total bilirubin 1.7 mg/dl, ammonia 147 μg/ml, lactate 2.5 mmol/L, and prothrombin time 17.8 s. The meticulous search for the underlying causes of acute liver injury was non-revealing. With volume repletion, mannitol use, and urine alkalization coupled with avoidance of nephrotoxic and hepatotoxic agents, his clinical features and laboratory abnormality completely resolved in 3 weeks. Despite rarity, severe rhabdomyolysis and/oracute liver injury as a potential life-threatening condition requiring urgent management may occur in high-altitude hypobaric hypoxia.

## Introduction

Mountain climbing for recreation or enhancement of physical performance is getting popular worldwide. In mountains higher than 2,500 m above sea level, high-altitude headache, central sleep apnea, and acute high-altitude illness (AHAI) may develop, especially in people without acclimatization. AHAI, including acute mountain sickness (AMS), high-altitude cerebral edema (HACE), and high-altitude pulmonary edema (HAPE), usually occurs after hours to 4 days because of poor adjustment to an oxygen-decreased environment (1, 2). Headache, gastrointestinal symptoms, fatigue/weakness, and dizziness/light-headedness are four major symptoms of AMS. In severe cases, AMS may progress to HACE with neurologic deficits such as conscious change and/or HAPE featuring dyspnea, cough, frothy sputum, and cyanosis. Slow ascent, premedication (pharmacological prophylaxis), and pre-acclimatization can prevent AHAI (2).

Acute high-altitude illness is a systemic maladaptation to hypoxia with predominant involvement of the brain and lungs. However, other organs such as the heart, liver, kidney, and muscles also participate in the response to hypoxia and thus may be affected by high-altitude hypoxia (2). For example, increased heart rate and cardiac output, metabolic adjustment and increased capillary density in skeletal muscles, decreased renal blood flow, transient kidney dysfunction, increased production of bicarbonate and erythropoietin in the kidneys, and central vein congestion in the hepatic lobule are notable in humans and animals (2–4). Nevertheless, organ injuries during AHAI were rarely reported and even less-appreciated, leading to misdiagnosis and inappropriate management. In this case report, we described a healthy man who presented with severe rhabdomyolysis, acute liver damage, and kidney injury after 3 days of high mountain climbing rapidly recognized and managed with prompt recovery.

## Case presentation

**FIGURE 1:**
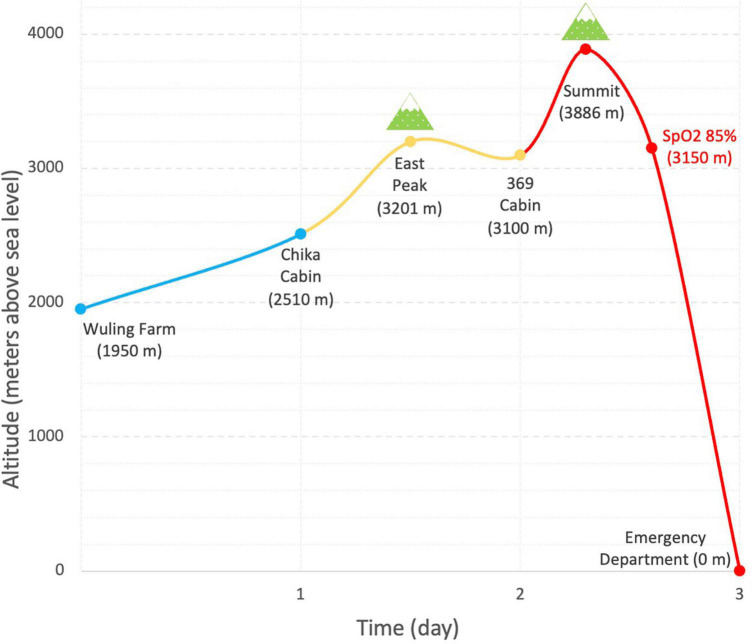
Ascent profile and time of climbing Snow Mountain (Xueshan). Blue, yellow, and red curves denote the ascent on the first, second day, and third days, respectively.

A 53-year-old healthy mountain climber presented to the emergency room with generalized malaise, diffuse muscle pain, dizziness, and tea-colored urine. He has climbed seventeen high mountains (all of them are higher than 3,000 m above sea level) in Taiwan and has experienced sleep disturbance occasionally during previous climbs. Because of the COVID-19 pandemic, he had discontinued mountain-climbing for a year. As the epidemic slowdown, he started to climb Snow Mountain in a group of 9 people 3 days before being admitted to the emergency department. He didn’t take pre-medication such as acetazolamide. As shown in Figure 1, he ascended from Wuling Farm (1,950-m altitude above sea level) to Chika Cabin (2,510 m) on the first day. He slept over at 369 Cabin (3,100 m) on the second day. When reaching the summit of Snow Mountain (3,886 m), he felt shortness of breath, mild headache, nausea, mild weakness, and moderate dizziness (2018 Lake Louise Acute Mountain Sickness Score: 5) (5). He descended immediately with notice of desaturation on oximeter (85%) when approaching 369 Cabin (3,150 m) and was sent to the hospital right away. He denied insect bites and ingestion of wild plants or raw meals. His personal and past histories were unremarkable. His liver and kidney functions (AST 25 IU/L, ALT 33 IU/L, creatinine 1.1 mg/dl, estimated GFR 74.4%) were normal 1 week prior to the mountain climb.

On physical examination, his consciousness was alert. His blood pressure was 114/74 mmHg, heart rate was 66 beats/min, and body temperature was 36.8°C. His respiratory rate was 17 bpm, and blood oxygen saturation on pulse oximetry was 98% without oxygen support. In addition, muscle pain and soreness, diminished skin turgor, dry oral mucosa, and tea-colored urine were noted. Urine analysis showed positive occult blood without red blood cells. As shown in Table 1, pertinent laboratory data revealed creatine kinase (CK) 33,765 IU/L, aspartate aminotransferase (AST) 2,882 IU/L, alanine aminotransferase (ALT) 2,259 IU/L, lactate 2.5 mmol/L, serum urea nitrogen (BUN) 26 mg/dl, and creatinine 1.5 mg/dl.

**TABLE 1:** Laboratory data at emergency department.

Test	Result	Reference range
Hemoglobin, g/dL	14.4	12–16
White blood cell count, /μL	14,970	3700–11000
Platelet count, ×10^3^/μL	198	150–400
Creatine phosphokinase, IU/L	33,765	39–308
Aspartate aminotransferase, IU/L	2,882	8–31
Alanine aminotransferase, IU/L	2,259	0–41
Serum urea nitrogen, mg/dL	26	10–25
Creatinine, mg/dL	1.5	0.7–1.4
Serum bicarbonate, mEq/L	26.8	22.0–27.0
Serum phosphate, mg/dL	1.9	2.7–4.5
Serum calcium, mg/dL	8.2	8.6–10.2
Total bilirubin, mg/dL	1.7	0.3–1.0
Ammonia, μg/mL	147	3.5–5.0
Lactate, mmol/L	2.5	0.5–2.2
PT, second	17.8	11–15

**FIGURE 2:**
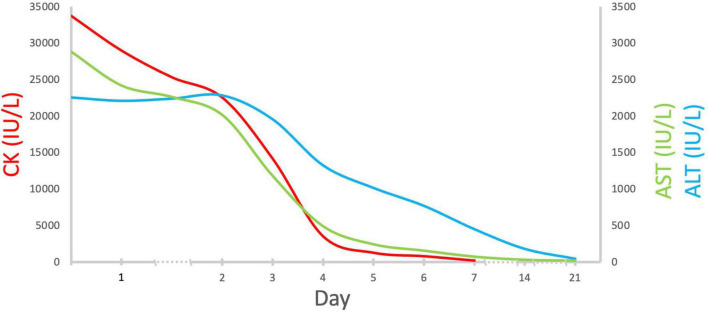
Serial changes in serum CK, AST, and ALT levels. Abbreviations: CK, creatine kinase; AST, aspartate aminotransferase; ALT, aspartate aminotransferase.

Aside from severe rhabdomyolysis with acute kidney injury (KDIGO stage 1, McMahon risk score 3) (6, 7), the disproportionally high level of AST and ALT (CK/AST ratio 11.7, CK/ALT ratio 14.9) was indicative of a coexisting acute liver damage. A detailed survey for the underlying causes of acute liver damage and rhabdomyolysis after obtaining informed consent was non-revealing. A further examination revealed an elevated serum ammonia 147 ug/dl, total bilirubin 1.7 mg/dl, and prolongation of prothrombin time, 17.8 s. Abdominal sonography demonstrated increased liver density and renal cortical echogenicity. With volume repletion, mannitol, and sodium bicarbonate administration, his serum creatinine declined to baseline (1.1 mg/dl) and even at a lower level (0.7 mg/dl) 1 day and 1 week later, respectively. His clinical features and rhabdomyolysis rapidly resolved (Figure 2) with an uneventful hospital course. His liver function completely normalized in 3 weeks.

## Discussion

This healthy mountain climber exhibited severe rhabdomyolysis, acute kidney injury, and acute liver damage after a sojourn at a high altitude. All the possible causes of the coexisting rhabdomyolysis and liver dysfunction such as drugs, heat stroke, infection, autoimmune, and genetic diseases were not identified except for hypobaric hypoxia (Table 2; 8–15). To the best of our knowledge, hypobaric hypoxia-related severe rhabdomyolysis, acute kidney injury, and acute liver damage have not been well-reported.

**TABLE 2:** Etiologies of concurrent rhabdomyolysis and liver dysfunction.

Etiologies	
Hypoxia	
Sepsis	
Drugs	Alcohol, antibiotics (daptomycin), cocaine, heroin, lipid-lowering agent (statin), phencyclidine
Heat stroke	Classical, exertional
Infection	β-hemolytic Streptococci, Bacillus cereus, influenza A/B, enteroviruses, Epstein-Barr virus, parainfluenza, herpes simplex virus, adenovirus, SARS-CoV-2
Intoxication	Wasp venom
Autoimmune diseases	Dermatomyositis, polymyositis
Genetic diseases	Glycogen storage diseases, mitochondrial chain disorder

It is well-known that AHAI mainly consists of AMS, HACE, and HAPE, and represents maladaptation of the brain and lungs to hypobaric hypoxia. However, the effect of hypobaric hypoxia is systemic. In this patient, skeletal muscles, liver, and kidney are affected after climbing up to 3,886 m altitude. At such high altitude, the expected resting oxygen saturation is around 82∼88% (2). Hypoxia *per se* can result in rhabdomyolysis (16). Strenuous exercise during high mountain climbing requires more oxygen consumption to generate energy, thus worsening hypoxic stress in striated muscles despite compensatory responses, such as angio-adaption and metabolic adjustment, that vary individually (2). Accordingly, both hypobaric hypoxia and strenuous exercise are most likely responsible for his severe rhabdomyolysis, defined as CK greater than 5000 IU/L, carrying a higher risk of complications (17).

Apart from severe rhabdomyolysis, the disproportionate ratio of CK/liver function profiles (AST, ALT) also indicates the presence of liver injury. It has been reported that a CK/AST and CK/ALT ratio lower than the cutoff point of 15–20 is helpful in diagnosing concurrent hepatic injury in rhabdomyolysis. The ratio of CK/AST (11.7) and CK/ALT (14.9) in this patient was less than 15, pointing to the presence of acute liver injury (18). The *R* value (the ratio of ALT to alkaline phosphatase) was 9.8, suggestive of predominant hepatocellular rather than cholestatic hepatitis. The prolonged prothrombin time and elevated serum ammonium also reflected severe liver damage in this patient. The much higher AST than ALT without alcoholic liver disease and rapid reduction of both aminotransferases without hypotension or hypoxia after admission highly favored ischemic hepatitis (19, 20). Since there are no other identifiable causes for ischemic hepatitis, acute hepatocellular injury caused by hypobaric hypoxia is plausible.

The acute kidney injury found in this patient could be related to volume depletion, myoglobin-related oxidative stress, tubular obstruction, and acute liver injury (17, 20). His relatively decreased baseline estimated GFR might make him more vulnerable to acute kidney injury. However, the rapid recovery of renal function with volume repletion, mannitol, and sodium bicarbonate administration indicated that his acute kidney injury is highly related to ischemic rather than toxic acute tubular necrosis (ATN). According to the McMahon risk prediction score for kidney failure or mortality in rhabdomyolysis, higher risk of severe acute kidney injury or mortality was notable once the score is higher than 5 (7). In this patient, his risk score was only 3, suggestive of the favorable kidney outcome as shown by the rapid improvement of kidney function after the treatment.

It is well-established that the presence of multiple organ dysfunctions is associated with much higher morbidity and mortality. The mortality rate is approximately up 10–40% when there are two to four organs involved (21). Because of systemic involvement in AHAI, all high-altitude visitors should take the danger of hypobaric hypoxia seriously. Pre-travel consultation, slow ascent, and pre-acclimatization are keys to avoid maladjusted responses. Pre-travel consultation includes evaluating the traveler’s underlying health conditions, identifying those who are sensitive to hypoxia, and making contingency plans for possible medical issues at high altitudes. As the major risk factor of acute altitude illness, ascent rate can be measured by sleeping elevation. Above 3,000 m altitude, sleeping elevation should not increase more than 500 m per night and rest days should be arranged every 3–4 days. Lastly, pre-acclimatization consists of exposure to hypoxia or staying at intermediate altitudes prior to the planned journey (2). A pharmacologic prophylaxis such as acetazolamide is highly recommended for the people with a history of AHAI or medical illness which is vulnerable to hypobaric hypoxia. Oxygen supplement, hyperbaric bag or chamber, and rapid descent are still the key treatments for the impending or developed AHAI (2).

The strength of this case is to remind clinicians that hypobaric hypoxia not only leads to AHAI but also causes systematic damages such as liver and kidney injuries, which should draw more attention. Also, certain tools are applied for evaluating the complications of the liver and kidneys in severe rhabdomyolysis. Further studies to investigate the detailed pathogenesis of hypobaric hypoxia affecting human body are still warranted.

In conclusion, this case highlighted the fact that severe rhabdomyolysis, acute liver damage, and kidney injury requiring early recognition and prompt management may develop in AHAI. Given its higher risk of multiple organ involvements with increased morbidity and mortality, being well-prepared with an insight into the danger of hypobaric hypoxia is important to ensure a safe high-altitude mountain climb.

## Data availability statement

The original contributions presented in the study are included in the article/supplementary material, further inquiries can be directed to the corresponding author.

## Ethics statement

The studies involving human participants were reviewed and approved by Ethics Committee on Human Studies at Tri-Service General Hospital in Taiwan. The patients/participants provided their written informed consent to participate in this study. Written informed consent was obtained from the individual(s) for the publication of any potentially identifiable images or data included in this article.

## Author contributions

Y-CY authored the manuscript and prepared the figures and tables, with contribution from C-CC and S-HL. C-CC revised the manuscript. S-HL supervised the manuscript. All authors contributed to the article and approved the submitted version.
